# Calbindin 2-specific deletion of arginase 2 preserves visual function after optic nerve crush

**DOI:** 10.1038/s41419-023-06180-6

**Published:** 2023-10-10

**Authors:** Syed A. H. Zaidi, Zhimin Xu, Tahira Lemtalsi, Porsche Sandow, Sruthi Athota, Fang Liu, Stephen Haigh, Yuqing Huo, S. Priya Narayanan, David J. R. Fulton, Modesto A. Rojas, Abdelrahman Y. Fouda, Robert W. Caldwell, Ruth B. Caldwell

**Affiliations:** 1 Vascular Biology Center, Augusta University, Augusta, GA 30912 USA; 2 Department of Medicine, Augusta University, Augusta, GA 30912 USA; 3 James and Jean Culver Vision Discovery Institute, Augusta University, Augusta, GA 30912 USA; 4 Department of Pharmacology and Toxicology, Augusta University, Augusta, GA 30912 USA; 5 Research Division, Charlie Norwood VA Medical Center, Augusta, GA 30904 USA; 6 Department of Cellular Biology and Anatomy, Augusta University, Augusta, GA 30912 USA; 7 Program in Clinical and Experimental Therapeutics, College of Pharmacy, University of Georgia, Augusta, GA 30912 USA; 8 Department of Pharmacology and Toxicology, University of Arkansas for Medical Sciences, Little Rock, AR 72205 USA

**Keywords:** Retina, Cell death in the nervous system

## Abstract

We previously found that global deletion of the mitochondrial enzyme arginase 2 (A2) limits optic nerve crush (ONC)-induced neuronal death. Herein, we examined the cell-specific role of A2 in this pathology by studies using wild type (WT), neuronal-specific calbindin 2 A2 KO (Calb2^cre/+^ A2 ^f/f^), myeloid-specific A2 KO (LysM^cre/+^ A2^f/f^), endothelial-specific A2 KO (Cdh5^cre/+^ A2^f/f^), and floxed controls. We also examined the impact of A2 overexpression on mitochondrial function in retinal neuronal R28 cells. Immunolabeling showed increased A2 expression in ganglion cell layer (GCL) neurons of WT mice within 6 h-post injury and inner retinal neurons after 7 days. Calb2 A2 KO mice showed improved neuronal survival, decreased TUNEL-positive neurons, and improved retinal function compared to floxed littermates. Neuronal loss was unchanged by A2 deletion in myeloid or endothelial cells. We also found increased expression of neurotrophins (BDNF, FGF2) and improved survival signaling (pAKT, pERK1/2) in Calb2 A2 KO retinas within 24-hour post-ONC along with suppression of inflammatory mediators (IL1β, TNFα, IL6, and iNOS) and apoptotic markers (cleavage of caspase3 and PARP). ONC increased GFAP and Iba1 immunostaining in floxed controls, and Calb2 A2 KO dampened this effect. Overexpression of A2 in R28 cells increased Drp1 expression, and decreased mitochondrial respiration, whereas ABH-induced inhibition of A2 decreased Drp1 expression and improved mitochondrial respiration. Finally, A2 overexpression or excitotoxic treatment with glutamate significantly impaired mitochondrial function in R28 cells as shown by significant reductions in basal respiration, maximal respiration, and ATP production. Further, glutamate treatment of A2 overexpressing cells did not induce further deterioration in their mitochondrial function, indicating that A2 overexpression or glutamate insult induce comparable alterations in mitochondrial function. Our data indicate that neuronal A2 expression is neurotoxic after injury, and A2 deletion in Calb2 expressing neurons limits ONC-induced retinal neurodegeneration and improves visual function.

## Introduction

Retinal ganglion cells (RGC) are neurons whose axons travel through the optic nerve to connect the retina to the brain. Therefore, injury to the optic nerve can lead to RGC death and permanent vision loss. This damage can occur due to pathological conditions such as glaucoma, traumatic optic neuropathy, or optic neuritis. However, the specific mechanisms by which this pathology promotes neuronal cell death are not well understood. Therefore, therapeutic strategies to limit the damage and promote repair are lacking. A commonly used method to study the process of optic nerve damage and the resultant loss of RGC and bystander retinal neurons is the mouse model of optic nerve crush (ONC) [1].

Our research has been focused on examining the function of the arginase pathway in retinal neurovascular injury. Arginase is the ureohydrolase enzyme that converts L-arginine to urea and ornithine. Arginase has two isoforms, Arginase 1 and Arginase 2, and both have been implicated in the pathogenesis of various brain and retinal diseases [2]. We have previously shown that the cytosolic isoform, Arginase 1, has a neuroprotective role in models of ischemia-reperfusion (IR) injury and retinopathy of prematurity (ROP) [3, 4]. Our laboratory has also shown that the expression of the mitochondrial isoform of arginase, arginase-2 (A2), increases early in mouse models of ONC injury as well as in models of ischemia/reperfusion injury (I/R) and retinopathy of prematurity (ROP) [5–8]. We have also demonstrated that global deletion of A2 can reduce neurovascular degeneration in all of these models. However, the cell-specific involvement of A2 is as yet unclear.

In our current project, we analyzed the specific role of neuronal expression of A2 in ONC injury. We used a neuronal-specific Cre driver to delete A2 in Calbindin 2 (Calb2) expressing neurons. Our study showed that Calb2-A2 deletion improved neuronal survival after ONC. Further, Calb2-specific A2 deletion increased survival signaling, suppressed the ONC-induced increases in inflammatory mediators and apoptotic markers, and preserved retinal structure and visual function after ONC. Additionally, our studies using R28 retinal neurons showed that A2 overexpression induces Drp1 expression and promotes mitochondrial dysfunction similar to glutamate-induced excitotoxicity. Our data indicate that A2 expression increases in Calb2-expressing neurons after injury, and blockage of A2 activity can be a therapeutic strategy for preventing retinal neuronal cell death.

## Results

### A2 expression is increased in retinal neurons after ONC

**Fig. 1:**
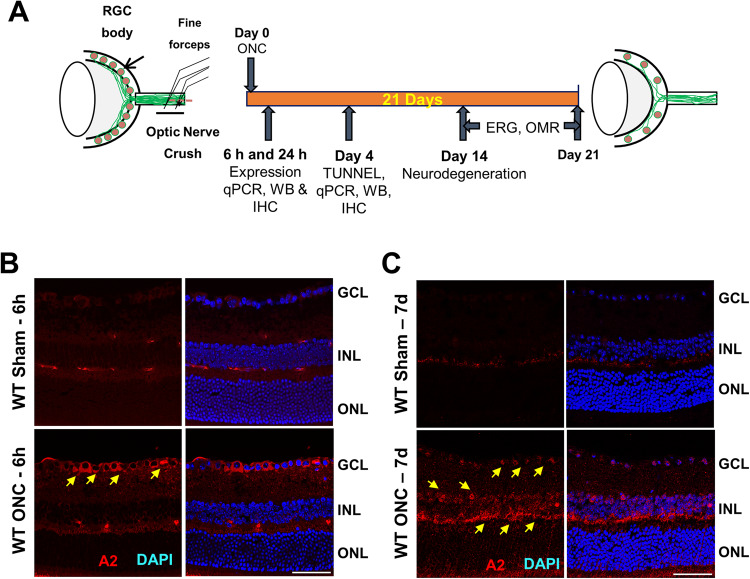
Arginase 2 expression is increased in retinal neurons after optic nerve crush. **A** Schematic representation of time-points for analyses after ONC. **B**, **C** Immunofluorescence imaging of WT retinal sections for A2 immunostaining (in red) showed increases in A2 in response to ONC at 6 h and 7 days after injury (arrows). *n* = 4. Scale bar = 40 μm.

Our previous analyses showed that ONC injury induces a marked increase in A2 levels in whole retinal extracts within six hours after injury. In order to assess the specific cell types that could be involved in this increase, we examined A2 localization in retina sections using immunofluorescence imaging techniques. Studies using wild-type C57BL/6 J (WT) sham control mice showed weak immunoreactivity for A2 in cells of the ganglion cell (GCL) and inner nuclear layer (INL) as well as in vessel-like structures in the inner and outer plexiform layers. In contrast, retinas prepared after ONC showed a robust increase in A2 expression in GCL neurons at 6 h post-injury which persisted for 7 days (Fig. 1B, C). Further, on day 7 post-ONC, A2 immunoreactivity was also strongly increased in cells of the INL (Fig. 1C).

### Calbindin 2 td tomato imaging of retinal neurons

**Fig. 2:**
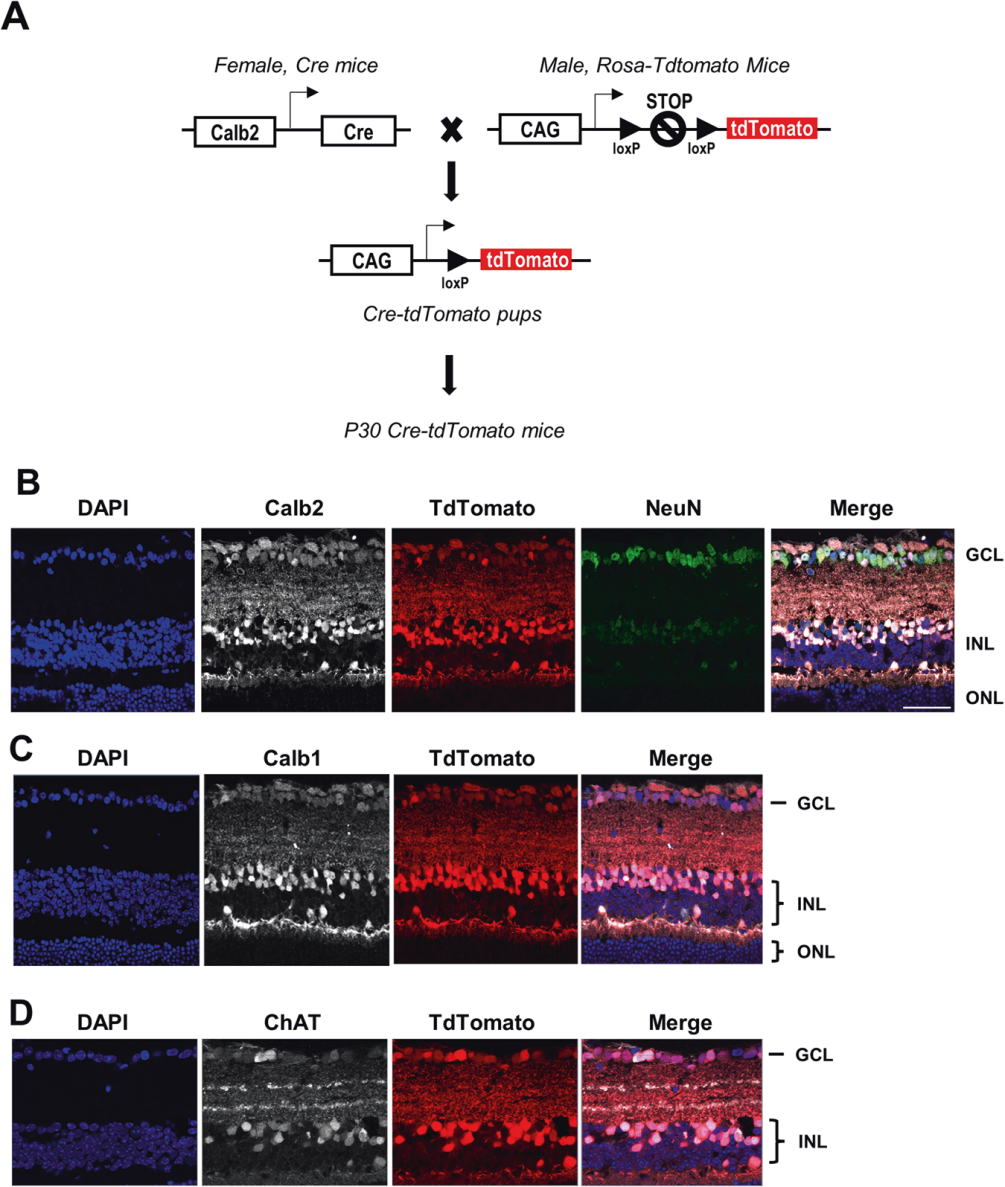
Calbindin2 tdTomato imaging of retinal neurons. **A** Breeding strategy for cre-lox-mediated expression of the tdTomato fluorescent protein. The recombination allows for excision of the STOP codon, thus leading to tdTomato expression under the CAG (CMV enhancer, chicken beta-Actin promoter, and rabbit beta-Globin splice acceptor site) promoter in cells expressing Calb2-cre. **B** Immunofluorescence imaging of Calb2/td retina sections showed strong colocalization of tdTomato expression with Calbindin2 (Calb2) and NeuN in GCL and INL. **C**, **D** Immunofluorescence imaging of Calb2/td retina sections showed strong colocalization of tdTomato expression in Calbindin1 (Calb1)-positive amacrine and horizontal cells as well as in Choline acetyltransferase (ChAT)-positive amacrine cells. *n* = 3. Scale bar = 40 μm.

Examination of retina sections from Calb2/td mice showed strong expression of tdTomato protein in neurons of the GCL and INL (Fig. 2B). Colocalization studies showed strong expression in GCL neurons of tdTomato along with the neuronal marker, NeuN, and Calbindin2. Also, many Calbindin 1-positive amacrine and horizontal cells and some choline acetyltransferase-positive amacrine cells were also positive for tdTomato protein (Fig. 2C, D).

### Calbindin 2-specific A2 deletion is neuroprotective against ONC

**Fig. 3:**
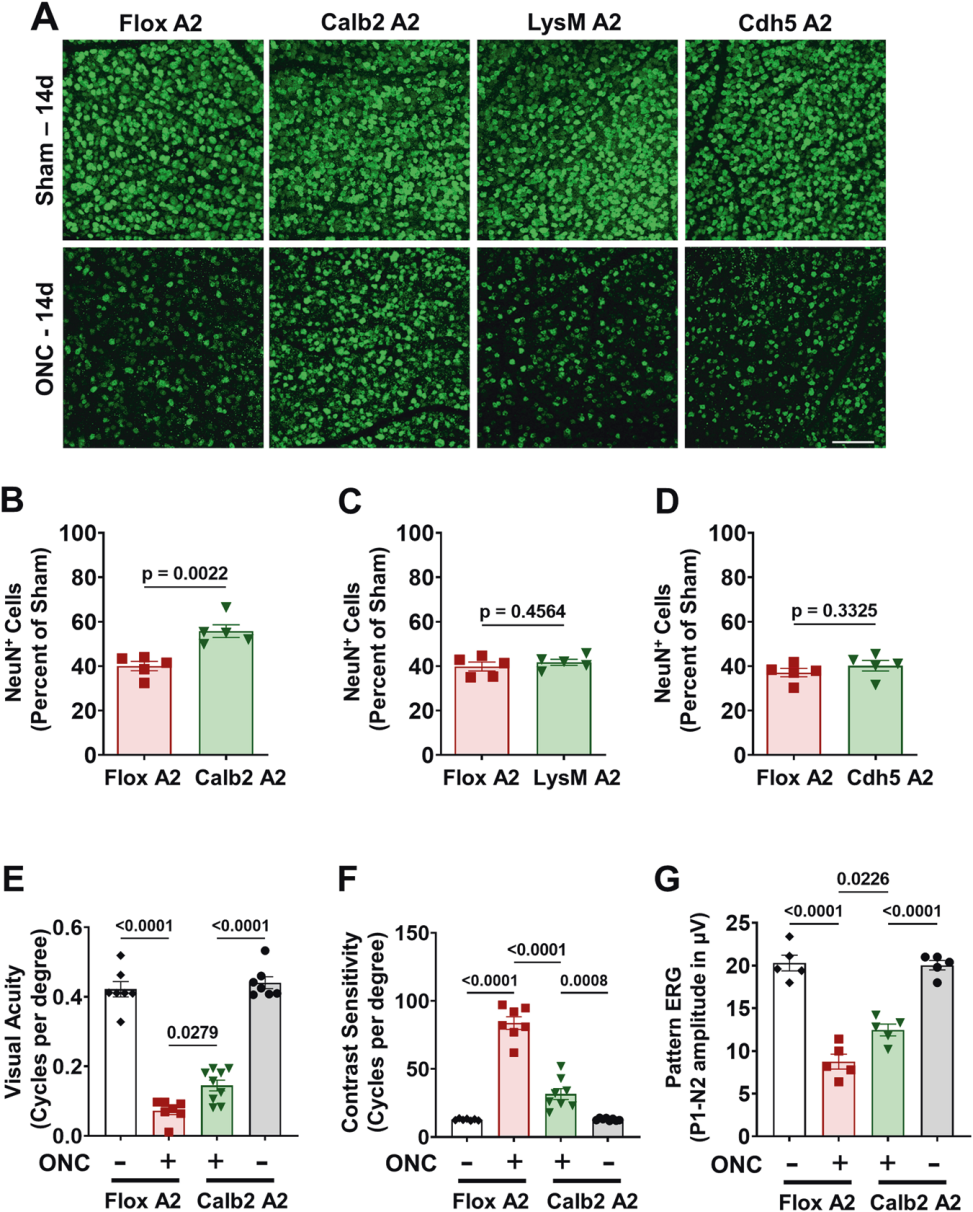
Calbindin 2-specific A2 deletion is neuroprotective and preserves visual function after ONC. Neuronal-specific (Calb2^cre/+^ A2^f/f^), myeloid-specific (LysM^cre/+^ A2^f/f^) and endothelial cell-specific (Cdh5^cre/+^ A2^f/f^) A2 KO, and floxed littermate mice were subjected to ONC injury and retinas were collected for analysis on day 14. **A** Confocal imaging of retinal flatmounts labeled with the neuronal marker, NeuN and (**B**) quantification showing a significant increase in NeuN positive cells in Calb2 A2 KO retina compared to A2 floxed control. **C**, **D** Myeloid-specific or endothelial cell-specific A2 KO did not show protection against ONC-induced neuronal loss. Data are presented as percent of A2^f/f^ sham. *n* = 5; scale bar = 40 μm. Data are presented as mean ± SEM. Retinal functional changes were measured by OptoMotry responses (OMR) and Pattern ERG in Calb2 A2 KO and floxed littermate mice at day 14 post-ONC. **E**, **F** Both visual acuity and contrast sensitivity were improved in Calb2 A2 KO mice as compared with the A2 floxed controls. *n* = 7–8. Data are presented as mean ± SEM. **G** Quantification of P1-N2 amplitude in the Pattern ERG showed a marked improvement in the Calb2 A2 KO mice as compared with the floxed controls. *n* = 5. Data are presented as mean ± SEM.

Our previous studies showed that global A2 deletion protects retinal neurons against ONC injury, but the specific cell types were not determined [5]. Given that A2 expression is markedly increased in retinal neurons of the GCL and INL after ONC and that many of these neurons express high levels calbindin 2, we reasoned that A2 expression in calbindin 2-positive neurons could be involved in the ONC-induced injury. To test this, we crossed A2 floxed mice with Calb2-Cre mice to specifically delete A2 from GCL neurons, amacrine cells and horizontal cells. We subjected these calbindin 2-specific A2 KO (Calb2 A2 KO) mice to ONC, and quantified survival of GCL neurons by immunostaining of retinal whole mounts with NeuN at day 14 post-injury. This analysis showed that ONC induced a significant loss of NeuN positive GCL neurons in A2 floxed control mice, and that the neuronal loss was reduced in Calb A2 KO mice (*p* = 0.0022; *n* = 5) (Fig. 3A, B). We also determined the effect on the neuronal injury of A2 deletion in myeloid cells and endothelial cells (EC). For this, we crossed A2 floxed mice with LysM cre and Cdh5 cre mice to generate mice lacking A2 in myeloid-derived cells (M-KO) or endothelial cells (E-KO), respectively. Neuronal cell loss was not altered by A2 KO in either the myeloid cells (Fig. 3A, C) or endothelial cells (Fig. 3A, D) as compared with the floxed control mice.

### Calbindin 2-specific A2 deletion preserves visual function

To access the nature of neuroprotection against ONC damage provided by A2 deletion in Calb2 expressing neurons, we monitored retinal function in A2 floxed and Calb2 A2 KO mice at day 14 post-ONC. First, we measured changes in visual acuity and contrast sensitivity using the optokinetic response tracking technique. As shown in Fig. 3E, F both visual acuity and contrast sensitivity were significantly improved in Calb2 A2 KO animals as compared with A2 floxed mice at day 14 post injury (*p* < 0.05; *n* = 7–8) suggesting that by A2 deletion in Calb2 neurons preserves visual function against ONC. Previous studies have shown that ganglion cell loss due to ONC has no effect on the light-adapted flash ERG a- and b-waves. However, pattern ERG responses were found to be significantly impaired following ONC injury [9]. Similar to previously published reports, ONC led to significant reductions in amplitude for both P1 and N2 (P1-N2) waves in A2 floxed control animals at day 14, compared to sham control. The declines in pattern ERGs were significantly prevented in Calb2 A2 KO animals (*p* = 0.0226; *n* = 5; Fig. 3G).

### Calbindin 2-specific A2 deletion promotes survival signaling after ONC

**Fig. 4:**
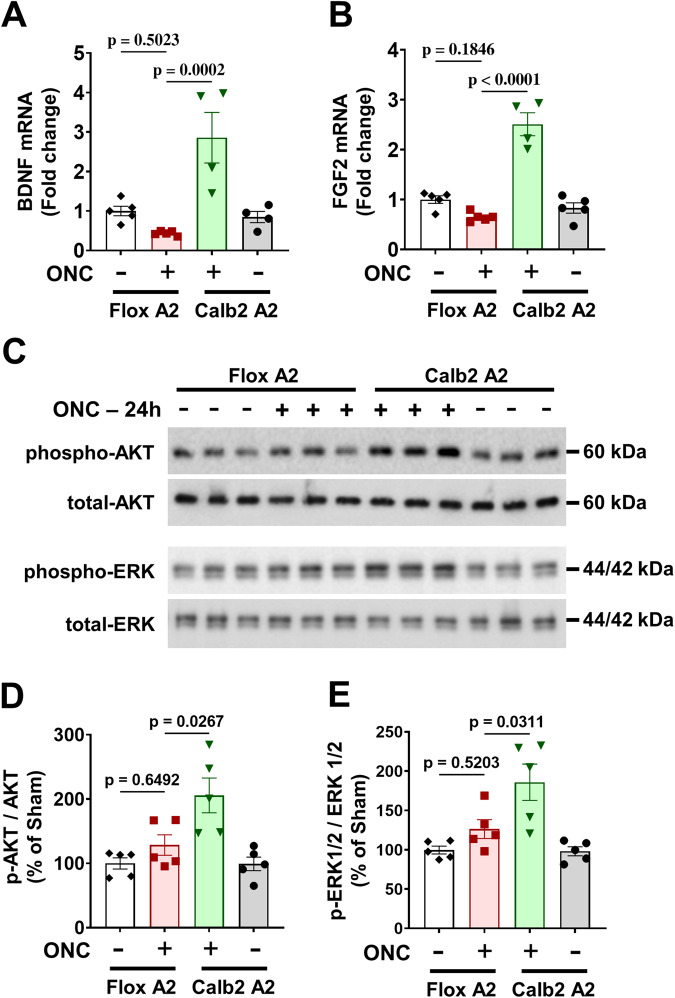
Calbindin 2-specific A2 deletion increases neurotrophic factor expression and promotes pro-survival signaling. **A**, **B** Quantitative RT-PCR showed a trend towards suppression of the neurotrophic factors BDNF and FGF2 at 24 h after ONC in the A2 floxed control retinas, whereas both were markedly increased in the Calb2 A2 KO retinas. *n* = 4. Data are presented as mean ± SEM. **C** Western blotting and (**D**, **E**) quantification of Akt and ERK1/2 phosphorylation in whole retinal lysates at 24 h after injury showed significant activation of pro-survival signaling in the Calb2 A2 KO retinas as compared with the A2 floxed controls. *n* = 5. Data are presented as mean ± SEM.

We have previously shown that the improved survival of GCL neurons after ONC in the A2 KO mice is associated with increased BDNF expression and activation of downstream pro-survival signaling via the mitogen-activated protein kinases extracellular signal-regulated kinases 1 and 2 (ERK1 and 2) [5]. Moreover, studies have shown that fibroblast growth factor 2 (FGF2) gene delivery stimulates growth of retinal ganglion cell axons after acute optic nerve injury [10]. We therefore determined the effect of Calb2 A2 KO on expression of BDNF and FGF2 following ONC. RT-PCR analysis showed that mRNA levels of BDNF and FGF2 were markedly increased after ONC in the Calb2 A2 KO retinas (Fig. 4A, B). Both BDNF and FGF2 tended to decrease in A2 floxed retinas following ONC, compared with the sham controls. Interestingly, neither was altered in Calb2 A2 KO sham control retinas as compared with the A2 floxed shams (*p* < 0.001; *n* = 4; Fig. 4A, B).

Studies have shown that BDNF activates ERK1/2 and protein kinase B (PKB/Akt) via a phosphatidyl-inositol-3′-kinase (PI-3-K)-dependent mechanism [11]. We therefore tested phosphorylation-mediated activation of Akt and ERK1/2 in whole lysates from A2 floxed and Calb2 A2 KO retinas by Western blotting. This analysis showed a significant increase in the phosphorylation of Akt (*p* = 0.0267; *n* = 5; Fig. 4C, D) and ERK1/2 (*p* = 0.0311; *n* = 5; Fig. 4C, E) in Calb2 A2 KO retinas after ONC when compared with floxed control ONC retinas. Our data also showed a slight, but non-significant, increase in the phosphorylation of both Akt and ERK1/2 in A2 floxed retinas compared to the sham control at 24 h post ONC (*p* > 0.05; *n* = 5; Fig. 4C–E).

### Calbindin 2-specific A2 deletion limits ONC-induced cell death

**Fig. 5:**
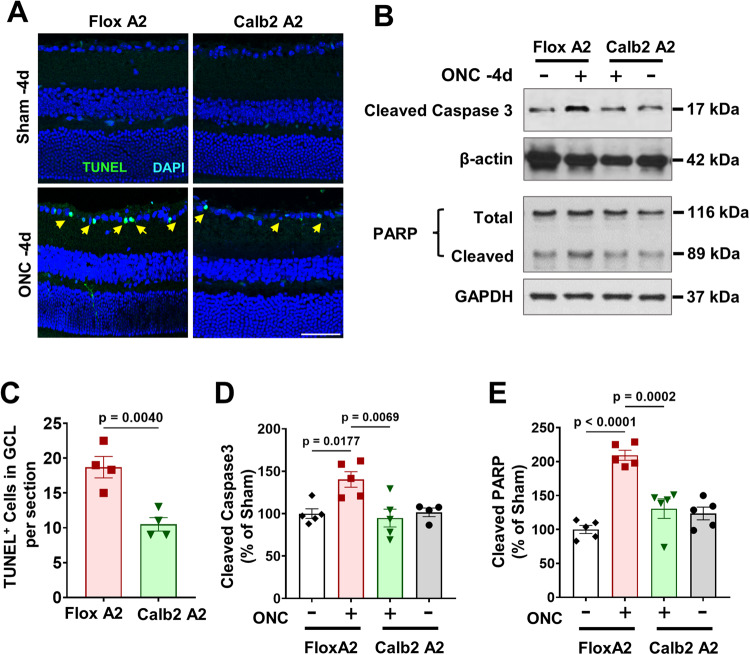
Calbindin 2-specific A2 deletion limits ONC-induced cell death. **A** Retinal cell death was measured by TUNEL immunolabeling of retina cross sections in Calb2 A2 KO and floxed control mice at day 4 post-ONC. **C** Quantification showed increased TUNEL positive cells in the GCL (arrows) at 4 days after ONC. Calb2 A2 KO mice also showed fewer TUNEL positive RGC neurons compared to A2 floxed controls. *n* = 4. Scale bar = 40 μm. Data are presented as mean ± SEM. **B** Western blotting and (**D**, **E**) quantification on whole retinal lysates at 5 days after ONC showed significant reductions in levels of cleaved Caspase 3 and PARP in the Calb2 A2 KO retinas as compared with the A2 floxed controls. *n* = 5. Data are presented as mean ± SEM.

Deprivation of neurotrophic factors such as brain derived neurotrophic factor (BDNF) has been reported in models of RGC death [12]. Apoptosis by DNA damage has been described as the one of the mechanisms of GCL neuronal cell death after ONC injury [13]. To ascertain the role of neuronal cell A2 in ONC-induced DNA damage, we performed TUNEL staining in A2 floxed and Calb2 A2 KO retina sections at 4 days post-injury. We found ONC-induced significant DNA damage in A2 floxed retinas compared to sham control. However, Calb2 A2 KO retinas showed fewer TUNEL positive GCL neurons (*p* = 0.004; *n* = 4) compared to littermate floxed control (Fig. 5A, C). Progressive DNA damage in dying neurons eventually leads to activation of caspase 3 and caspase 3-mediated degradation of poly ADP-ribose polymerase (PARP), markers of programmed neuronal death. Activation of caspase 3 requires proteolytic processing into activated p17 fragments. Western blotting data showed significant activation/cleavage of both caspase 3 and PARP in A2 floxed retinas at day 4, post-injury, and Calb2 A2 KO significantly decreased the intensities of bands of cleaved caspase3 (*p* = 0.0069; *n* = 5; Fig. 5B, D) and PARP (*p* = 0.0002; *n* = 5; Fig. 5B, E) proteins.

### Calbindin 2-specific A2 deletion reduces activation of Müller cells and microglia/macrophage and prevents inflammation after ONC

**Fig. 6:**
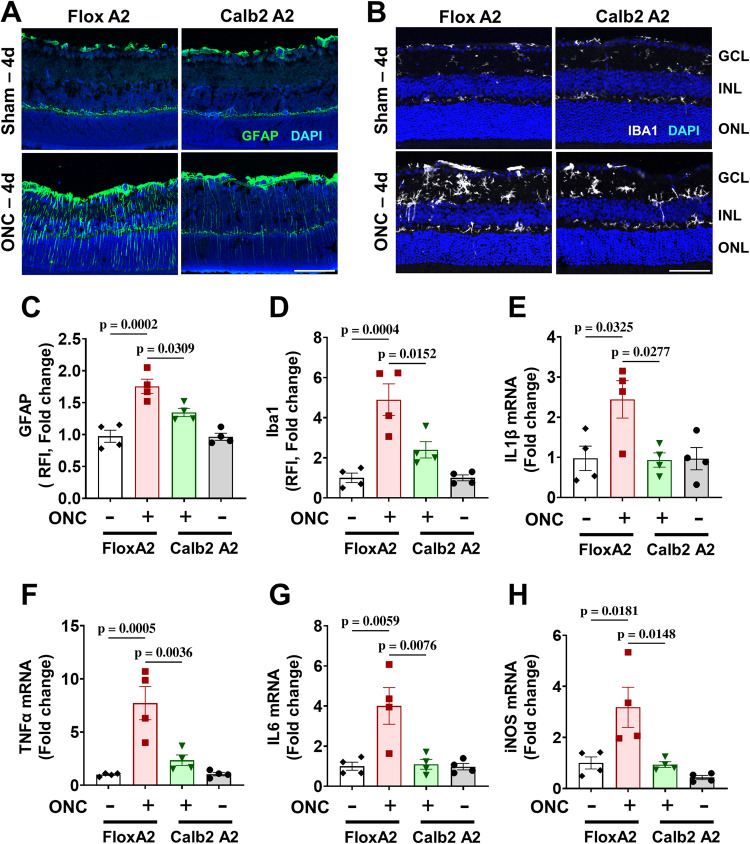
Calbindin 2-specific A2 deletion reduces ONC-induced activation of Müller cells and microglia/macrophage and prevents inflammation. Immunolabeling for (**A**) GFAP and (**B**) Iba1 in retina cross sections together with quantification (**C**, **D**) showed increased activation of Müller cells and microglia/macrophage, respectively, in A2 floxed control retinas at 4 days after ONC. This was ameliorated in the Calb2 A2 KO retinas. *n* = 4. Scale bar = 40 μm. Data are presented as mean ± SEM. **E**–**H** Quantitative RT-PCR showed upregulation of proinflammatory mediators - IL1β, TNFα, IL6, and iNOS at 5 days after ONC in the A2 floxed control retinas. Calb2 A2 KO significantly blocked this increase. *n* = 4. Scale bar = 50 μm Data are presented as mean ± SEM.

To determine whether Calb2 A2 KO is sufficient to reduce glial and microglial/macrophage activation, we immunostained A2 floxed and Calb2 A2 KO retina sections for GFAP and Iba1 at 4 days post-injury. A2 floxed retinas showed significant increases in immunoreactivity for both markers (*p* = 0.0002; *n* = 4; Fig. 6A, C) and Iba1 (*p* = 0.0004; *n* = 4; Fig. 6B, D), suggesting an increase in activation of Müller cells and retinal microglia/macrophages, respectively. Calb2-specific A2 deletion blocked this increased activation as confirmed by decreased fluorescence intensity for both GFAP (*p* = 0.0309; *n* = 4; Fig. 6A, C) and Iba1 (*p* = 0.0152; *n* = 4; Fig. 6B, D), as compared to A2 floxed ONC retinas.

Prolonged activation of retinal microglia/macrophages has been shown to induce inflammation and retinal neurodegeneration [14]. We performed RT-PCR to determine the changes in the mRNA expression of pro-inflammatory markers interleukin-1β (IL-1β), tumor necrosis factor-α (TNF-α), interleukin-6 (IL-6), and iNOS based on their potential involvement in neurodegenerative diseases. RT-PCR results indicated elevated levels of these mediators in A2 floxed ONC retinas at day 4. The upregulation of these molecules was significantly inhibited in Calb2 A2 KO ONC retinas (Fig. 6E–H).

### A2 overexpression in neuronal cells induces mitochondrial dysfunction

**Fig. 7:**
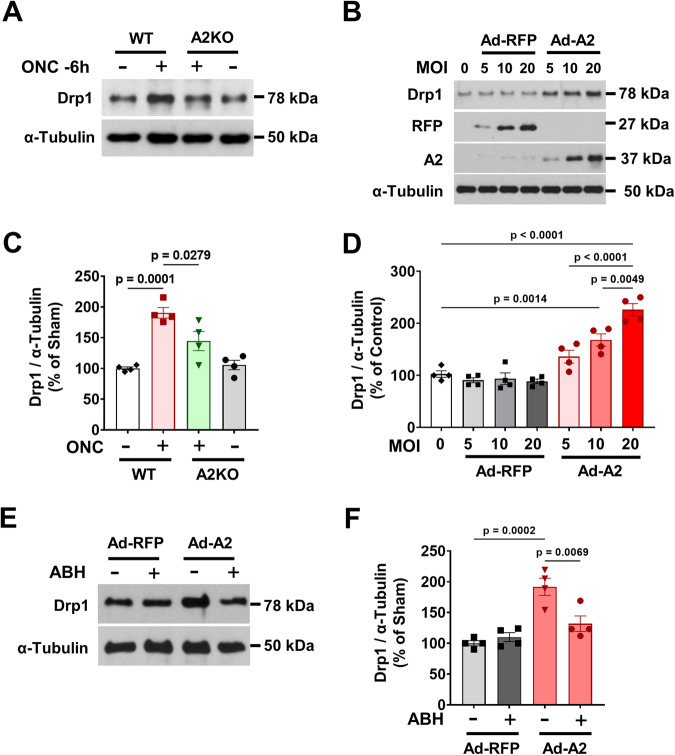
A2 expression induces Drp1-mediated mitochondrial dysfunction in vivo and in vitro. **A**, **C** WT and A2KO mice were subjected to ONC for 6 h. Western blotting and quantification of Drp1 in whole retinal lysates at 6 h after injury showed significant increase in Drp1 in the WT retinas compared with the sham. Global deletion of A2 significantly suppressed ONC-induced Drp1 upregulation. *n* = 4. Data are presented as mean ± SEM. **B**, **D** Retinal neuronal R28 cells were differentiated and transduced with Ad-RFP or Ad-A2 at increasing multiplicities of infection (5, 10, 20 MOI). Western blot analyses with quantification showed increased expression of Drp1 with increasing expression of A2 compared to R28 transduced with increasing RFP expression. *n* = 4. Data are presented as mean ± SEM. **E**, **F** A2 or RFP overexpressing R28 cells were treated with 1 µM of ABH or vehicle for 16 h. Western blot analyses with quantification showed ABH treatment significantly reduced Drp1 expression in A2 overexpressing cells. *n* = 4. Data are presented as mean ± SEM.

Because A2 is localized to the mitochondria, we tested whether A2 expression during injury contributes to mitochondrial dysfunction. Mitochondrial dysfunction was assessed by analyzing expression of the mitochondrial fission protein-dynamin related protein 1 (Drp1) in WT and global A2KO mice subjected to ONC. Retinas were analyzed for Drp1 expression at 6-hour post-ONC. Drp1 was significantly increased in WT retinas, and deletion of A2 reducedits expression (Fig. 7A, C). Next, we determined the effect of overexpression of A2 on Drp1 expression in R28 retinal neuronal cells in vitro. R28 cells were transduced with adenoviral vectors containing A2 or RFP for 6 h at a 5, 10 and 20 multiplicities of infection (MOI). Overexpression of A2 significantly increased Drp1 levels compared with the RFP controls (Fig. 7B, D). Furthermore, the increase in Drp1 was significantly inhibited by treatment with the global arginase inhibitor 2(S)-amino-6-boronohexanoic Acid (ABH) (Fig. 7E, F).

**Fig. 8:**
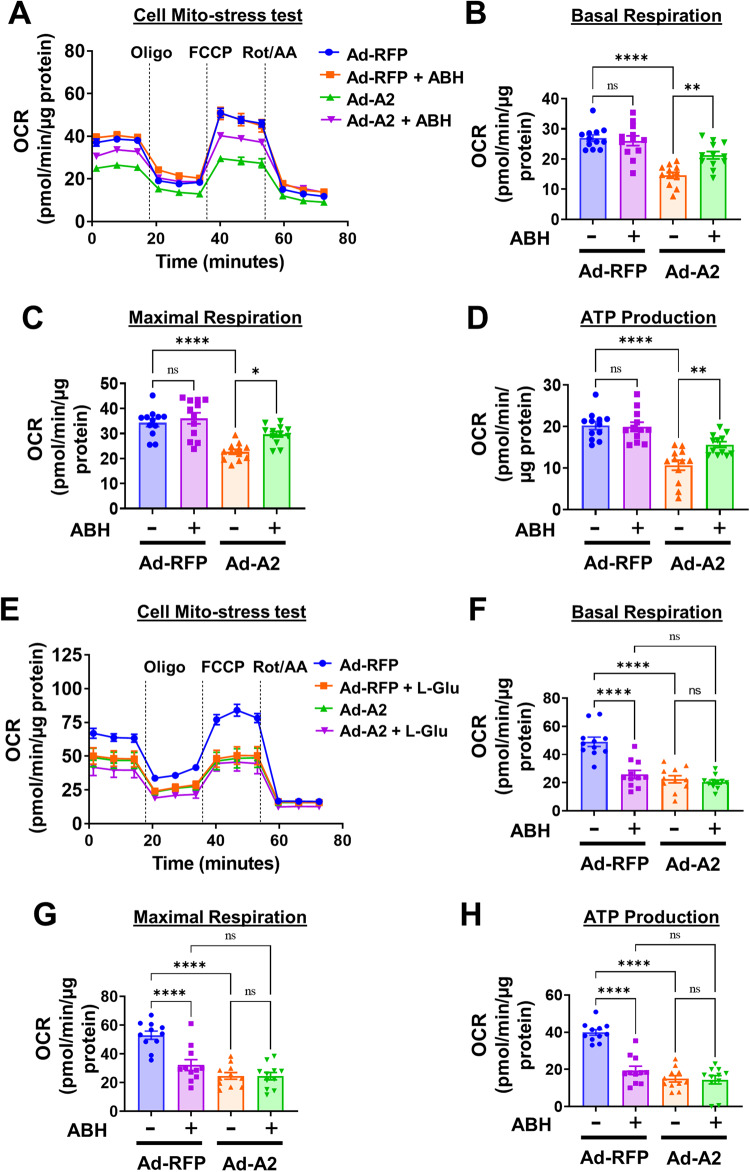
A2 overexpression impairs mitochondrial respiration in vitro. A2 or RFP were overexpressed in retinal neuronal R28 cells, followed by treatment with vehicle or 1 µM ABH or 5 mM glutamate or vehicle for 16 h. Oxygen consumption rate (OCR) was measured using Seahorse Xfe96. **A**–**D** A2 overexpression decreased OCR, along with decreased basal respiration, maximal respiration, and ATP production. Treatment with ABH in A2 overexpressing cells significantly improved these parameters. *P < 0.05; **P < 0.01; ****P < 0.0001. *n* = 12. Data are normalized to protein content and presented as mean ± SEM. **E**–**H** A2 overexpression and/or L-glutamate treatment decreased OCR. Basal respiration, maximal respiration, and ATP production showing a decreased OCR with A2 overexpression and/or L-glutamate treatment group compared to RFP. ****P < 0.0001. *n* = 11. Data are normalized to protein content and presented as mean ± SEM.

Next, we measured mitochondrial respiration using Seahorse XFe flux analyzer. A2 or RFP was overexpressed at MOI of 20 in R28 cells, followed by treatment with 1 µM ABH or vehicle for 16 h. Following overexpression of A2, mitochondrial function was significantly impaired as shown by significant reductions in basal respiration, maximal respiration, and ATP production as compared to the RFP control (Fig. 8A–D). However, treatment with ABH significantly improved mitochondrial OCR response and basal respiration, maximal respiration, and ATP production. We also examined mitochondrial function in a model of glutamate-induced neurotoxicity using the Seahorse technique. Glutamate-mediated excitotoxicity has been shown to be a key mediator of neuronal injury in models in retina ischemia reperfusion injury and optic nerve crush [15, 16]. A2 or RFP were overexpressed in R28 rat neuronal cells, followed by treatment with 5 mM glutamate or vehicle for 16 h. Following overexpression of A2, mitochondrial function was significantly impaired as shown by significant reductions in basal respiration, maximal respiration, and ATP production as compared to the RFP control (Fig. 8E–H). Glutamate treatment also induced a significant decrease in these parameters, indicating mitochondrial dysfunction. The glutamate treatment of R28 cells transduced with A2 did not induce any further deterioration in their mitochondrial function, indicating that A2 overexpression or glutamate-induced neurotoxicity can induce comparable alterations in mitochondrial function.

## Discussion

Neurons in the RGC layer, like other neurons of the central nervous system, lack the ability to regenerate. Therefore, damage to the optic nerve, such as occurs in traumatic optic neuropathies and glaucoma, often results in RGC death and vision loss [17]. In addition, to such direct injury of the RGCs, studies have suggested an associated loss of sub-populations of displaced amacrine cells [18]). Several factors have been reported to be involved in the death of RGC neurons. These factors include but are not limited to mitochondrial dysfunction [19–21], ischemic/oxidative stress [19, 22, 23], lack of neurotrophins [11, 24], gliosis [25, 26], glutamate-induced excitotoxicity [27–30] and enhanced production of proinflammatory cytokines [31, 32]. Understanding the underlying cellular mechanisms responsible for progressive RGC neuronal degeneration in such optic neuropathies will help in identifying novel therapeutic targets and developing neuroprotective strategies that can mitigate this neuronal cell damage.

In this study, we focused on elucidating the role of the ureohydrolase enzyme arginase in ONC-induced loss of neurons in GCL and INL. Arginase hydrolyzes arginine to form ornithine and urea. Arginase has two isoforms: the cytosolic isoform arginase1 (A1) and the mitochondrial isoform arginase2 (A2). Our group’s previous research showed that A1 is neuroprotective, and that A1 deletion globally or in myeloid-derived cells aggravates retinal ischemia/reperfusion (I/R) injury and promotes an increase in inflammatory microglial/macrophage cells with increased expression of inducible nitric oxide synthase (iNOS) [3, 33]. We further showed that treatment with human recombinant A1 linked to polyethylene glycol (PegA1) protects against RGC death in mouse models of I/R and ONC by a mechanism involving decreased inflammation and upregulation of the reparative microglia/MΦ phenotype [3, 33].

In contrast to A1, A2 has a detrimental role in neuronal survival in retinal injury models of ONC, I/R, and OIR [5–8, 34]. Our previous study in the ONC model showed that A2 levels increase after ONC and that global deletion of A2 limits ONC-induced loss of RGC neurons along with decreased activation of microglia/Müller cells, reduced inflammation (iNOS, IL-1β), and increased pro-survival signaling via activation or BDNF/Akt/ERK [5]. Our studies in the I/R model indicated that A2 deletion limits neurovascular degeneration along with reducing formation of superoxide and peroxynitrite and suppressing activation of the p38 MAPK stress pathway [7, 8]. We also found that A2 deletion in the OIR model limits both neuronal and vascular injury via reductions in oxidative stress and inflammation [6, 34] However, the cell-specific role of A2 in these pathologies and the molecular mechanisms are not yet clear. Here we show that A2 expression is highly increased in GCL neurons of WT mice at 6 h post ONC (Fig. 1B). Further, A2 immunoreactivity in the GCL remains elevated at 7 days post-ONC and is also strongly increased in cells of the INL (Fig. 1C). However, we did not see any changes in A1 expression in retinal neurons upon ONC (data not shown).

In order to test the neuronal cell-specific role of A2 in ONC-induced injury we generated a transgenic mouse lacking A2 expression in calbindin2 (Calb2) expressing retinal neurons. Studies have shown that Calb2 is expressed by the majority of the RGC, amacrine cells, and horizontal cells in the murine retina [35, 36]. Our studies of Calb2-cre recombination using a tdTomato reporter mouse confirmed expression of Calb2 expression in neurons localized to the GCL and INL. Our data showed tdTomato protein in both GCL and INL neurons. Furthermore, the signal was co-localized with markers of ganglion, amacrine, and horizontal cells (Fig. 2B–D). Our analyses of neuronal survival in the Calb2 A2 KO and A2 floxed control using NeuN staining of retina flatmount at 14 days post-ONC showed a significant increase in numbers of NeuN positive neurons in Calb2 A2 KO mice as compared with A2 floxed control mice (Fig. 3A, B). In contrast, neuronal cell loss was not altered by A2 KO in either myeloid or endothelial cells (Fig. 3A, C, D). Our further analyses of visual function using optomotor testing and pattern electroretinography (PERG) showed significant improvements in visual acuity, contrast threshold, and ganglion cell function in Calb2 A2 KO mice compared to the A2 floxed controls at day 14 post-ONC (Fig. 3E–G). These data clearly indicate that Calb2-specific A2 deletion provides neuroprotection and improves vision function following ONC injury.

Deprivation of neurotrophic support and production of proinflammatory cytokines has been closely associated with reduced RGC survival [11, 24, 31, 32]. Our qRT-PCR data indicated that mRNA levels of BDNF and FGF2 were slightly, but not significantly, decreased in A2 floxed retinas following ONC as compared with the sham controls. However, both were markedly increased after ONC in the Calb2 A2 KO retinas as compared with the sham controls (Fig. 4A, B). Studies have shown that BDNF activates ERK1/2 and protein kinase B (PKB/Akt) via a phosphatidyl-inositol-3′-kinase (PI-3-K)-dependent mechanism [11]. We confirmed significant increases in the phosphorylation of Akt and ERK1/2 in Calb2 A2 KO retinas after ONC when compared with floxed control ONC retinas. (Fig. 4C–E).

Previous studies from our group have shown that global deletion of A2 limits neuronal death in mouse models of ONC, retinal I/R and OIR by reducing DNA fragmentation and caspase activation as shown by TUNEL labeling and cleavage of caspase 3 and PARP [5, 6, 8]. Here we show that Calb2-specific A2 KO significantly decreased the numbers of TUNEL positive GCL neurons compared to the floxed controls (Fig. 5A, C). Further, Calb2 A2 KO significantly decreased cleavage of caspase 3 and PARP proteins as compared with the A2 floxed mice at day 4, post-ONC (Fig. 5B, D, E).

We further examined whether the neuronal specific A2 deletion mitigates inflammation by analysis of glial and microglial/macrophage activation. Activation of Müller cells and microglia/macrophages has been reported following traumatic optic neuropathy and glaucomatous damage [37, 38]. We have shown that the expression of glial fibrillary acidic protein (GFAP) is increased upon activation of Müller cells and extends from the nerve fiber layer through the ONL. On the other hand, activated microglia/macrophage show enlarged processes and increased expression of ionized calcium binding adaptor molecule 1 (Iba1) [5]. Our current results showed that the ONC-induced neuronal loss in A2 floxed ONC retinas is accompanied by gliosis and microglia/macrophage activation as indicated by significant increases in both GFAP and Iba1 and that these effects were blocked by the Calb2-specific A2 deletion (Fig. 6A–D).

Because A2 is localized to the mitochondria, we also tested whether A2 expression during injury contributes to mitochondrial dysfunction. We have previously shown that the overexpression of A2, but not A1, induced mitochondrial dysfunction, as demonstrated by increased expression of the mitochondrial fission protein Drp1 and decreased mitochondrial respiration [7]. We tested whether ONC induces an increase in Drp1 expression in wild type and A2KO retinas. Drp1 levels increased in WT retinas at 6 h, post-ONC and global deletion of A2 attenuated this increase in Drp1 expression (Fig. 7A, C). Overexpression of A2 in the R28 retina neuronal cell line also resulted in increased Drp1 expression (Fig. 7B, D) and impaired mitochondrial function as compared to the RFP control (Fig. 8A–D), whereas inhibition by A2 by arginase inhibitor, ABH, decreased Drp1 expression (Fig. 7E, F) and improved mitochondrial function (Fig. 8A–D). Further, we also used glutamate treatment to mimic excitotoxicity in vitro. As expected, glutamate treatment also induced a significant impairment of mitochondrial function. Interestingly, the glutamate treatment in A2 overexpressing R28 cells did not induce any further deterioration in their mitochondrial function, indicating that A2 overexpression or glutamate insult can induce comparable alterations in mitochondrial function (Fig. 8E–H).

Overall, our data indicate that injury-induced upregulation of A2 is neurotoxic and that deletion of A2 in Calb2 expressing neurons limits ONC-induced retinal neurodegeneration and thereby improves visual function. Based on our findings, A2-mediated mitochondrial dysfunction is a probable mechanism involved in ONC-induced RGC layer neuronal cell death.

## Materials and methods

### Generation of transgenic mice

Animal work was done in agreement with the ARVO Statement for the Use of Animals in Ophthalmic and Vision Research. The studies were approved by the institutional animal care and use committee (Animal Welfare Assurance no. A3307-01). All surgeries were performed under anesthesia, and all efforts were made to minimize suffering. Wild type (WT) and global A2 knockout (A2KO) mice on C57BL6J background were generated in our animal colony as described previously [5, 8]. Published data indicate that Calb2 is expressed by the majority of the RGC, amacrine cells, and horizontal cells in the murine retina [35, 36]. Thus, we compared the effects of Calb2-specific A2 deletion on ONC-induced neuronal injury with those of myeloid or endothelial cell-specific A2 deletion. The A2 floxed (A2^f/f^) transgenic mice were obtained from Dr. Dmitri Firsov’s laboratory and then rederived in Jackson laboratories in a C57BL6J background [39]. A2 floxed mice were crossed with Cre-expressing transgenic mice under the control of Calb2 (Calb2-Cre; Jax stock #010774) to generate calbindin 2-specific A2 knockout (Calb2 A2 KO). The endothelial-specific and myeloid-specific A2 KO mice were generated as previously described [4, 7]. Briefly, A2 floxed mice were crossed with Cre-expressing transgenic mice under the control of either a VE-Cadherin promoter (Cdh5Cre; Jax stock# 006137) or lysozyme 2 promoter (LysMCre; Jax stock# 004781) to generate either endothelial-specific A2KO (E-A2KO) or myeloid-specific A2KO (M-A2KO) mice, respectively. We also examined Calb2-cre recombination in retinal tissue using a tdTomato reporter mouse. For this, we bred reporter tdTomato mouse (Jax stock# 007914) with the Calb2-Cre mice as outlined in our previous studies [40].

### Optic nerve crush

Controlled optic nerve crush (ONC) was performed as previously described [5]. Briefly, 10–12-week-old male mice were anesthetized with intraperitoneal ketamine (100 mg/kg) and xylazine (10 mg/kg). Before surgery, topical anesthetic (0.5% proparacaine HCl) was applied to the eyeball and surrounding area. The optic nerve of the left eye was exposed and crushed 1–2 mm from the eyeball with self-closing N7 forceps (Fine Science Tools, CA) for 3 s. Sham surgery was performed on the right eye by exposing the optic nerve without crush. Antibiotic ointment was applied to the eyes following the procedure and mice were injected subcutaneously with buprenorphine for analgesia. Eyes were harvested at different time points post-ONC, as indicated in Fig. 1A. A total of 8 animals were used in each group. We calculated the sample size based on our preliminary data and previous results in the lab. Power calculations reveal that when *n* = 8, the power value is >0.8 with 20% difference in the treatment effects and a probability of error of 0.05. Rare animals that develop cataract, swelling or reddening in eyes due to infection were excluded from the studies. No specific randomization scheme was used as neuronal-specific Calb2 A2 KO mice were compared to their littermate A2 floxed controls. Further, ONC injury was done in left eye and right served as sham control. However, during surgery, mice were randomly picked without revealing their genotype.

### Wholemount retina and GCL neuronal cell counting

Neuronal degeneration was assessed at 14 days after ONC injury as previously described [5]. Eyeballs were enucleated and fixed overnight in 4% paraformaldehyde in PBS at 4 °C. The retinas were dissected into whole mounts and incubated with the neuronal marker anti-NeuN antibody (Millipore, Cat. # MAB377, Billerica, MA, 1:300 dilution) overnight at 4 °C and then incubated with Alexa fluor 488 conjugated secondary antibody (Invitrogen, Cat. # A-11059, Carlsbad, CA, 1:600 dilution). Four z-stack images were taken in the midperiphery region of each retina wholemount using an inverted confocal microscope (LSM 780; Carl Zeiss, Thornwood, NY), and NeuN-positive cells were counted using ImageJ software. Data were analyzed by investigators blinded to the group allocation. Data are represented NeuN-positive cell numbers in the GCL of the ONC eyes as a percent of the A2^f/f^ sham control.

### Immunofluorescence imaging

Enucleated eyeballs were fixed in PFA and cryoprotected. Retina cryostat sections were permeabilized in Triton -X 100 (1% in PBS) for 10 min and blocked in 10% normal goat serum containing 1% BSA for 1 h. Sections were then incubated overnight in primary antibodies at 4 °C. The next day, the sections were incubated at room temperature for 1 h in fluorescent conjugated secondary antibodies (Invitrogen, Carlsbad, CA) and mounted with a DAPI-containing medium (Vector Laboratories, Cat. #1400). The following primary antibodies were used: A2 (Abcam, cat. # ab228700, 1:300), Calbindin 2 (Sigma, cat. # MAB1568, 1:500), Calbindin 1 (Sigma, cat. # C9848, 1:500), Choline acetyltransferase (Sigma, cat. # AB144P, 1:500), GFAP (Dako, Cat. # Z0334, 1:400), Iba1 (Wako, cat. # 019-19741), 20X and 40X Images were taken in a different region of each retina section using an inverted confocal microscope (LSM 780; Carl Zeiss, Thornwood, NY). Fluorescence intensities were quantified using FIJI ImageJ2 software, and data are represented as relative fluorescent intensities (RFI) in ONC retinas compared to the A2^f/f^ sham control.

### Quantitative RT-PCR

Total RNA extraction from mouse retinas was performed using RNAqueous 4PCR total RNA isolation kit (Invitrogen, Carlsbad, CA, US) per the manufacturers’ instruction. cDNA was prepared from 0.5 μg RNA as a template for reverse transcription using M-MLV reverse transcriptase (Invitrogen). qRT PCR was performed on an ABI 7500 Real-Time PCR System (Applied Biosystems, Foster City, CA) using SYBR Green master mix (Invitrogen) and the respective gene-specific primers, BDNF, FGF2, IL1β, TNFα, IL6, and iNOS. The sequences of these primers were published previously [4, 33, 41]. The relative gene expression is calculated using the comparative threshold cycle (ΔΔCt) method against the hypoxanthine phosphoribosyl-transferase (HPRT) internal control. Expression levels for all genes are reported as fold change relative to the A2 floxed sham control.

### Protein extraction and western blotting

Protein extracts were prepared by homogenizing the retina or R28 cells using a handheld micro-grinder in RIPA lysis buffer (Millipore Sigma) supplemented with protease and phosphatase inhibitors (Complete Mini and phosSTOP Roche Applied science, Indianapolis, IN) [42]. Lysates were then centrifuged at 14,000 rpm for 12 min at 4 °C, and total proteins in the supernatant were quantified by Bradford Protein Assay (BioRad, Hercules, CA). 10–20 µg of total protein were separated on 10–12% sodium dodecyl sulfate-polyacrylamide gel electrophoresis and then transferred to nitrocellulose membrane. The membrane was blocked in 2% BSA in TBST (Tris-buffered saline with 0.1% Tween-20) for 1 h at room temperature, followed by overnight incubation with primary antibody at 4 °C. The next day, membranes were washed with TBST and incubated with horseradish peroxidase-linked secondary antibody (Cell Signaling, 1:2000) for 1 hour. The bands were detected and captured using ECL (enhanced chemiluminescence), Western blotting substrate (Thermofisher Scientific), and Biorad Versadoc imaging system (Biorad, Hercules, CA). The following primary antibodies were used: PARP (Cell Signaling, cat. #9542), p-ERK1/2 (Cell signaling, cat. #4370), ERK1/2 (Cell Signaling, cat. #4695 S), pAkt (Cell Signaling, cat. #4060), Akt (Cell signaling, cat. #4691) and cleaved capspase3 (Cell Signaling, cat. #9664), Drp1 (Cell signaling, cat, #8570). Membranes were reprobed with β-actin (Sigma-Aldrich, cat. # A5441) or GAPDH (Meridian Bioscience, cat. # H86504M) to ensure equal protein loading. Band intensities of each protein of interest were measured by densitometry as arbitrary units and normalized with β-actin or α-Tubulin or GAPDH as an internal loading control.

### R28 retinal neuronal like cells

R28 cells were purchased from Kerafast, Inc. Boston, MA. R28 cells from passages 67 to 70 were cultured in DMEM with low glucose (Sigma, cat# D5523) supplemented with 10% fetal calf serum (Cytiva HyClone, cat# CSH3007303) and 1% penicillin-streptomycin (Gibco, cat# 15070063), 7.5% sodium bicarbonate (Sigma, cat# S8761), 1% MEM non-essential amino acids (Gibco, cat# 11130051) as described previously [33]. R28 cells were differentiated overnight in complete medium supplemented with 250 μM pCPT-cAMP (Sigma, cat# C3912) on laminin-coated plates. The next day, cells were transduced with adenoviral vectors containing either A2 or RFP for 6 h at 5, 10, and 20 multiplicities of infection (MOI), as previously described [7]. The final MOI of 20 was chosen according to previously published studies and preliminary experiments [7]. After 6 h, the media was changed to normal complete growth media for 24 h. The following day, cells were treated with vehicle or 1 µM ABH or 5 mM L-Glutamate for 16–18 h and subjected to Western blot and Seahorse XFe flux analysis.

### Seahorse XFe96 Mito stress test

Seahorse XFe96 (Agilent, Santa Clara, CA) were used to evaluate mitochondrial dysfunction, as described previously [7]. Briefly, R28 cells were seeded in a differentiation medium (as described above) at a cell density of 20,000/well in the laminin-coated Seahorse cell culture 96 well plate in all the wells except A1, A12, H1 and H12 wells which were used as background wells. The day before the assay, the Seahorse media was prepared according to manufacturer’s instructions and supplemented with 2.5 mM glutamine (Gimini, West Sacramento, CA) and 5.5 mM glucose (Sigma, St. Louis, MO). Then, we ran the Mito stress test (Agilent, Catalogue # 103015-100, Santa Clara, CA) according to manufacturer’s instructions. The concentrations of the injections compounds used were as follow: Oligomycin (1 µM), FCCP (2 µM) and Rotenone/antimycin A (0.5 µM). The data were collected and analyzed using the Wave software (Agilent, Santa Clara, CA) and normalized to protein content.

### TUNEL (TdT-mediated dUTP nick end labeling) assay

TUNEL assay was performed to detect DNA damage/apoptotic cells on retinal sections using InSitu Cell Death Detection Kit (Millipore, cat. #S7110, Billerica, MA) based on the manufacturer’s recommendations. Counting and quantification were performed as previously described [5]. Briefly, two sections per animal were used to collect the images and the quantification of TUNEL positive cells was performed manually on the whole retinal section.

### Visual function assessment

#### Optomotor response

Visual acuity and contrast sensitivity were assessed using optokinetic response tracking (OKT) (Cerebral Mechanics, Inc., Lethbridge, AB, Canada) as described previously. Sine wave gratings were displayed along four computer screens revolving around a central stand. The optomotor reflex of the unrestrained mouse to the rotating vertical sine wave grating was recorded by manual tracking of reflexive head movements by an investigator blinded to their treatment status. To determine spatial frequency thresholds, an increasing stairstep algorithm was utilized starting at 0.042 cycles/deg (c/d) and fixed at maximum contrast. To determine the contrast threshold, the spatial frequency was set at 0.092 c/d, and a decreasing stairstep algorithm was utilized starting with maximum contrast. Readings for the left eye (clockwise head movement) and the right eye (counterclockwise head movement) were recorded.

#### Pattern electroretinogram (PERG) recording

Dark-adapted mice were anesthetized by xylazine and ketamine based on their body weight. Pupils were dilated with topical 0.5% tropicamide (Akorn, Lake Forest, IL, USA) and 2.5% phenylephrine HCL (Paragon BioTeck, Portland, OR USA). PERG recording of both eyes was performed simultaneously to investigate RGC function using the Celeris-Diagnosys system (Diagnosys, Lowell, MA, USA) according to a published protocol [43, 44]. Briefly, mice were placed on Celeris-Diagnosys system equipped with a feedback-controlled heating pad for maintaining animal core temperature at 37 °C, and small lubricant eye drop (GenTeal Lubricant Eye Gel, Alcon, Ft. Worth, TX) was applied before recording to prevent corneal dryness. A pattern ERG electrode was placed on the corneal surface, and visual stimulus generated by black and white alternating contrast reversing bars (spatial frequency 0.125 cycles/degree, a luminance of 50 cd s/m2, contrast 100%) was aligned with the projection of the pupil. The flash stimulator was placed on the contralateral eye as a reference electrode. Each pattern-ERG was an average of 600 sweeps at an interval of 1 s, with measurements made between a peak and adjacent trough of the waveform. The first positive peak in the waveform was designated as P1, and the second negative peak was N2. P1 was typically around 100 ms. The amplitude was measured from P1 to N2. The mean of the P1-N2 amplitude in the injured eye was compared to that in the sham control eye. The data were analyzed by the software Espion V6 (Diagnosys) and results are presented as averaged values of amplitude with the two eyes of each mouse.

### Statistical analyses

GraphPad Prism 9.4.1 was used to generate graphs and for statistical analyses. Data are presented as means ± SEM. Data were analyzed by investigators blinded to the group allocation. Genotype unmasking and group identity was performed after completion of experiments and analysis. One-way ANOVA with post-hoc Tukey multiple comparisons was used to analyze the statistical significance in studies of 3 or more groups. The significance of differences between 2 groups were determined by Student’s *t*-test. *P* < 0.05 was taken as significant. In each case, data was reviewed to see how well they fit the test assumptions.

### Supplementary information

Original Data file

## Data Availability

The data generated and/or analyzed during the current study are available on reasonable request. Correspondence and requests for materials should be addressed to SAHZ or RBC.

## References

[1] Templeton JP, Geisert EE (2012). A practical approach to optic nerve crush in the mouse. Mol Vis.

[2] Caldwell RW, Rodriguez PC, Toque HA, Narayanan SP, Caldwell RB (2018). Arginase: a multifaceted enzyme important in health and disease. Physiol Rev.

[3] Fouda AY, Xu Z, Shosha E, Lemtalsi T, Chen J, Toque HA (2018). Arginase 1 promotes retinal neurovascular protection from ischemia through suppression of macrophage inflammatory responses. Cell Death Dis.

[4] Fouda AY, Xu Z, Suwanpradid J, Rojas M, Shosha E, Lemtalsi T (2022). Targeting proliferative retinopathy: Arginase 1 limits vitreoretinal neovascularization and promotes angiogenic repair. Cell Death Dis.

[5] Xu Z, Fouda AY, Lemtalsi T, Shosha E, Rojas M, Liu F (2018). Retinal neuroprotection from optic nerve trauma by deletion of arginase 2. Front Neurosci.

[6] Narayanan SP, Xu Z, Putluri N, Sreekumar A, Lemtalsi T, Caldwell RW (2014). Arginase 2 deficiency reduces hyperoxia-mediated retinal neurodegeneration through the regulation of polyamine metabolism. Cell Death Dis.

[7] Shosha E, Fouda AY, Lemtalsi T, Haigh S, Fulton D, Ibrahim A (2021). Endothelial arginase 2 mediates retinal ischemia/reperfusion injury by inducing mitochondrial dysfunction. Mol Metab.

[8] Shosha E, Xu Z, Yokota H, Saul A, Rojas M, Caldwell RW (2016). Arginase 2 promotes neurovascular degeneration during ischemia/reperfusion injury. Cell Death Dis.

[9] Miura G, Wang MH, Ivers KM, Frishman LJ (2009). Retinal pathway origins of the pattern ERG of the mouse. Exp Eye Res.

[10] Sapieha PS, Peltier M, Rendahl KG, Manning WC, Di Polo A (2003). Fibroblast growth factor-2 gene delivery stimulates axon growth by adult retinal ganglion cells after acute optic nerve injury. Mol Cell Neurosci.

[11] Klocker N, Kermer P, Weishaupt JH, Labes M, Ankerhold R, Bahr M (2000). Brain-derived neurotrophic factor-mediated neuroprotection of adult rat retinal ganglion cells in vivo does not exclusively depend on phosphatidyl-inositol-3’-kinase/protein kinase B signaling. J Neurosci.

[12] Claes M, De Groef L, Moons L (2019). Target-derived neurotrophic factor deprivation puts retinal ganglion cells on death row: cold hard evidence and caveats. Int J Mol Sci.

[13] Li Y, Schlamp CL, Nickells RW (1999). Experimental induction of retinal ganglion cell death in adult mice. Invest Ophthalmol Vis Sci.

[14] Hu X, Zhao GL, Xu MX, Zhou H, Li F, Miao Y (2021). Interplay between Muller cells and microglia aggravates retinal inflammatory response in experimental glaucoma. J Neuroinflammation.

[15] Joo CK, Choi JS, Ko HW, Park KY, Sohn S, Chun MH (1999). Necrosis and apoptosis after retinal ischemia: involvement of NMDA-mediated excitotoxicity and p53. Invest Ophthalmol Vis Sci.

[16] Vorwerk CK, Zurakowski D, McDermott LM, Mawrin C, Dreyer EB (2004). Effects of axonal injury on ganglion cell survival and glutamate homeostasis. Brain Res Bull.

[17] Tham YC, Li X, Wong TY, Quigley HA, Aung T, Cheng CY (2014). Global prevalence of glaucoma and projections of glaucoma burden through 2040: a systematic review and meta-analysis. Ophthalmology.

[18] Akopian A, Kumar S, Ramakrishnan H, Viswanathan S, Bloomfield SA (2019). Amacrine cells coupled to ganglion cells via gap junctions are highly vulnerable in glaucomatous mouse retinas. J Comp Neurol.

[19] Jassim AH, Fan Y, Pappenhagen N, Nsiah NY, Inman DM (2021). Oxidative stress and hypoxia modify mitochondrial homeostasis during glaucoma. Antioxid Redox Signal.

[20] Williams PA, Harder JM, Foxworth NE, Cochran KE, Philip VM, Porciatti V (2017). Vitamin B3 modulates mitochondrial vulnerability and prevents glaucoma in aged mice. Science.

[21] Tribble JR, Otmani A, Sun S, Ellis SA, Cimaglia G, Vohra R (2021). Nicotinamide provides neuroprotection in glaucoma by protecting against mitochondrial and metabolic dysfunction. Redox Biol.

[22] Jassim AH, Nsiah NY, Inman DM (2022). Ocular hypertension results in hypoxia within glia and neurons throughout the visual projection. Antioxidants (Basel).

[23] Husain S, Abdul Y, Singh S, Ahmad A, Husain M (2014). Regulation of nitric oxide production by δ-opioid receptors during glaucomatous injury. PLoS One.

[24] Feng L, Puyang Z, Chen H, Liang P, Troy JB, Liu X. Overexpression of brain-derived neurotrophic factor protects large retinal ganglion cells after optic nerve crush in mice. eNeuro. 2017;4:ENEURO.0331-16.2016. 10.1523/ENEURO.0331-16.2016 PMC5240030 28101532

[25] Sun D, Moore S, Jakobs TC (2017). Optic nerve astrocyte reactivity protects function in experimental glaucoma and other nerve injuries. J Exp Med.

[26] Jassim AH, Coughlin L, Harun-Or-Rashid M, Kang PT, Chen YR, Inman DM (2019). Higher Reliance on glycolysis limits glycolytic responsiveness in degenerating glaucomatous optic nerve. Mol Neurobiol.

[27] Irnaten M, Duff A, Clark A, O’Brien C (2020). Intra-cellular calcium signaling pathways (PKC, RAS/RAF/MAPK, PI3K) in Lamina Cribrosa Cells in Glaucoma. J Clin Med.

[28] Guo X, Zhou J, Starr C, Mohns EJ, Li Y, Chen EP (2021). Preservation of vision after CaMKII-mediated protection of retinal ganglion cells. Cell.

[29] Verma M, Lizama BN, Chu CT (2022). Excitotoxicity, calcium and mitochondria: a triad in synaptic neurodegeneration. Transl Neurodegener.

[30] Fahrenthold BK, Fernandes KA, Libby RT (2018). Assessment of intrinsic and extrinsic signaling pathway in excitotoxic retinal ganglion cell death. Sci Rep.

[31] Echevarria FD, Formichella CR, Sappington RM (2017). Interleukin-6 deficiency attenuates retinal ganglion cell axonopathy and glaucoma-related vision loss. Front Neurosci.

[32] Husain S, Zaidi SAH, Singh S, Guzman W, Mehrotra S (2021). Reduction of neuroinflammation by delta-opioids via STAT3-dependent pathway in chronic glaucoma model. Front Pharmacol.

[33] Fouda AY, Eldahshan W, Xu Z, Lemtalsi T, Shosha E, Zaidi SA (2022). Preclinical investigation of Pegylated arginase 1 as a treatment for retina and brain injury. Exp Neurol.

[34] Narayanan SP, Suwanpradid J, Saul A, Xu Z, Still A, Caldwell RW (2011). Arginase 2 deletion reduces neuro-glial injury and improves retinal function in a model of retinopathy of prematurity. PLoS One.

[35] Kovacs-Oller T, Szarka G, Ganczer A, Tengolics A, Balogh B, Volgyi B (2019). Expression of Ca(2+)-binding buffer proteins in the human and mouse retinal neurons. Int J Mol Sci.

[36] Kovacs-Oller T, Szarka G, Tengolics AJ, Ganczer A, Balogh B, Szabo-Meleg E (2020). Spatial expression pattern of the major Ca(2+)-buffer proteins in mouse retinal ganglion cells. Cells.

[37] Gonzalez-Riquelme MJ, Galindo-Romero C, Lucas-Ruiz F, Martinez-Carmona M, Rodriguez-Ramirez KT, Cabrera-Maqueda JM (2021). Axonal injuries cast long shadows: long term glial activation in injured and contralateral retinas after unilateral axotomy. Int J Mol Sci.

[38] Zaidi SAH, Thakore N, Singh S, Guzman W, Mehrotra S, Gangaraju V (2020). Histone deacetylases regulation by delta-opioids in human optic nerve head astrocytes. Invest Ophthalmol Vis Sci.

[39] Ansermet C, Centeno G, Lagarrigue S, Nikolaeva S, Yoshihara HA, Pradervand S (2020). Renal tubular arginase-2 participates in the formation of the corticomedullary urea gradient and attenuates kidney damage in ischemia-reperfusion injury in mice. Acta Physiol (Oxf).

[40] Fouda AY, Xu Z, Narayanan SP, Caldwell RW, Caldwell RB (2020). Utility of LysM-cre and Cdh5-cre driver mice in retinal and brain research: an imaging study using tdTomato reporter mouse. Invest Ophthalmol Vis Sci.

[41] Zaidi SAH, Lemtalsi T, Xu Z, Santana I, Sandow P, Labazi L (2023). Role of acyl-coenzyme A: cholesterol transferase 1 (ACAT1) in retinal neovascularization. J Neuroinflammation.

[42] Shosha E, Qin L, Lemtalsi T, Zaidi SAH, Rojas M, Xu Z (2022). Investigation of retinal metabolic function in type 1 diabetic akita mice. Front Cardiovasc Med.

[43] Li L, Huang H, Fang F, Liu L, Sun Y, Hu Y (2020). Longitudinal morphological and functional assessment of RGC neurodegeneration after optic nerve crush in mouse. Front Cell Neurosci.

[44] Zaidi SAH, Guzman W, Singh S, Mehrotra S, Husain S (2020). Changes in class I and IIb HDACs by delta-opioid in chronic rat glaucoma model. Invest Ophthalmol Vis Sci.

