# Supplementary figures and images for: Calbindin 2-specific deletion of arginase 2 preserves visual function after optic nerve crush
Source: Cell Death Dis. 2023 Oct 10;14(10):661. doi: 10.1038/s41419-023-06180-6 (PMC10564748; doi:10.1038/s41419-023-06180-6)

Supplementary Figure 1

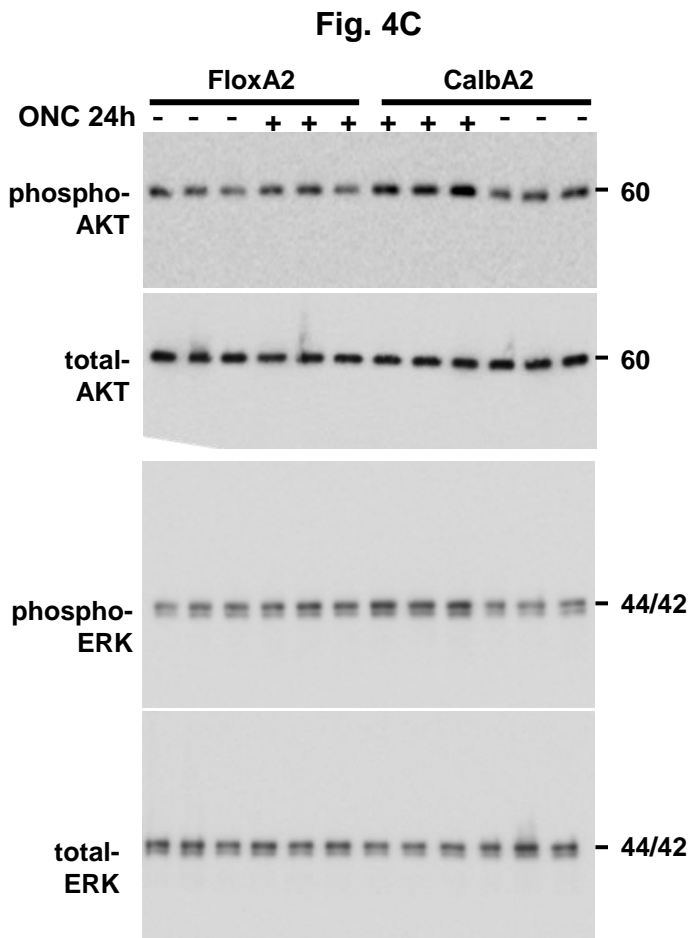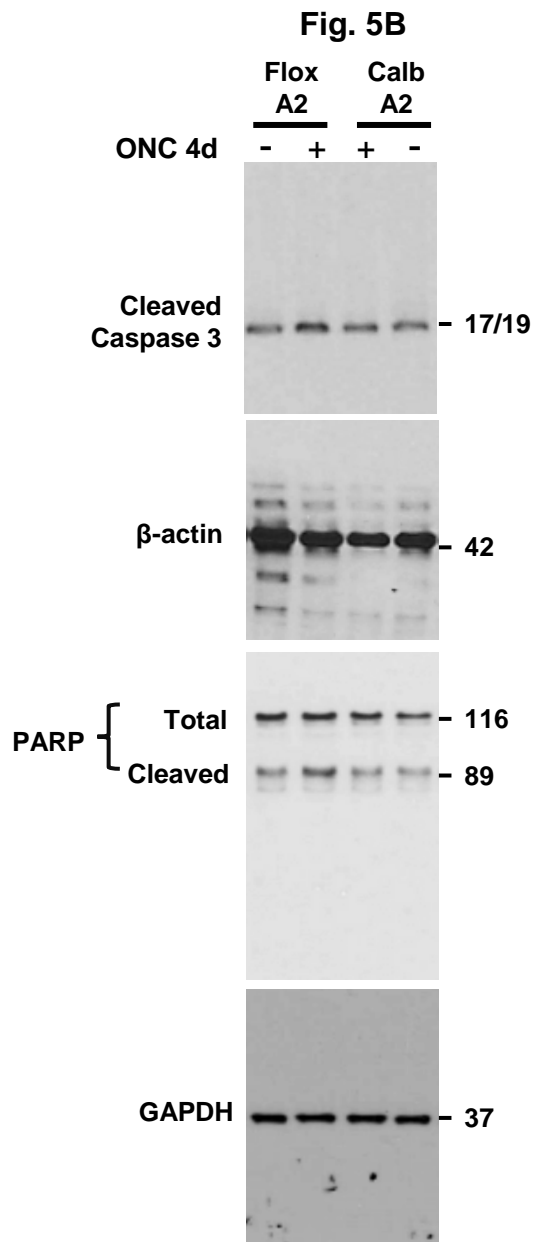

Supplementary Figure 2

Fig. 7A

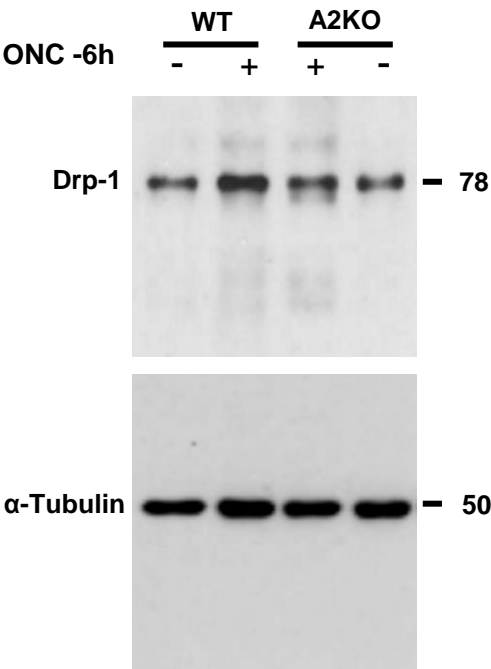

Fig. 7B

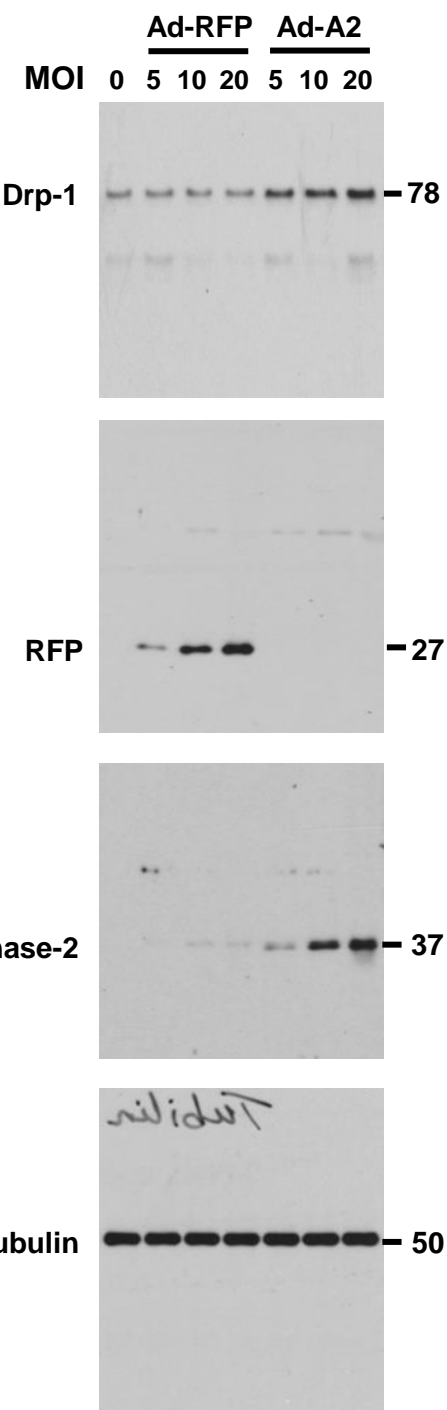

Fig. 7F

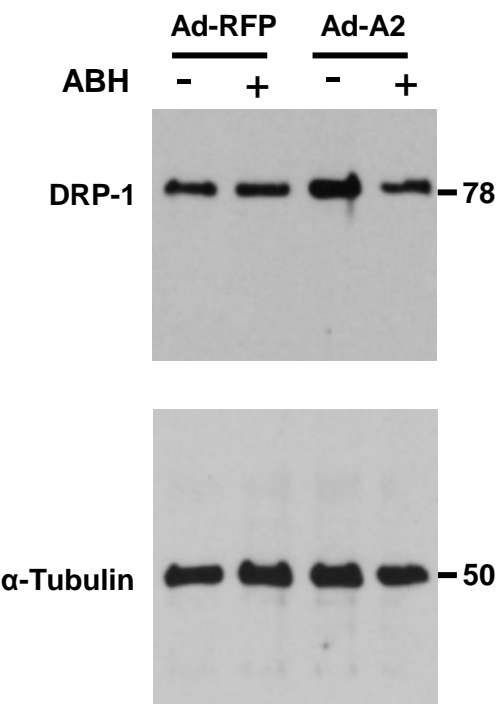

Supplement: Supplementary file 1 — Original Data file [file 41419_2023_6180_MOESM1_ESM.pdf]
